# Catheter radiofrequency ablation for arrhythmias under the guidance of the Carto 3 three-dimensional mapping system in an operating room without digital subtraction angiography

**DOI:** 10.1097/MD.0000000000011044

**Published:** 2018-06-22

**Authors:** Xingfu Huang, Yanjia Chen, Zheng Huang, Liwei He, Shenrong Liu, Xiaojiang Deng, Yongsheng Wang, Rucheng Li, Dingli Xu, Jian Peng

**Affiliations:** a Department of Cardiology; b Department of Anesthesiology, Nanfang Hospital, Southern Medical University, Guangzhou; c The Second People's Hospital of Jiedong District, Jieyang; d Guangning County People's Hospital, Zhaoqing, Guangdong Province, China.

**Keywords:** atrioventricular nodal re-entrant tachycardia, radiofrequency ablation, ventricular premature contracts, Wolff–Parkinson–White syndrome, zero-fluoroscopy

## Abstract

Several studies have reported the efficacy of a zero-fluoroscopy approach for catheter radiofrequency ablation of arrhythmias in a digital subtraction angiography (DSA) room. However, no reports are available on the ablation of arrhythmias in the absence of DSA in the operating room. To investigate the efficacy and safety of catheter radiofrequency ablation for arrhythmias under the guidance of a Carto 3 three-dimensional (3D) mapping system in an operating room without DSA. Patients were enrolled according to the type of arrhythmia. The Carto 3 mapping system was used to reconstruct heart models and guide the electrophysiologic examination, mapping, and ablation. The total procedure, reconstruction, electrophysiologic examination, and mapping times were recorded. Furthermore, immediate success rates and complications were also recorded. A total of 20 patients were enrolled, including 12 males. The average age was 51.3 ± 17.2 (19–76) years. Nine cases of atrioventricular nodal re-entrant tachycardia, 7 cases of frequent ventricular premature contractions, 3 cases of Wolff–Parkinson–White syndrome, and 1 case of typical atrial flutter were included. All arrhythmias were successfully ablated. The procedure time was 127.0 ± 21.0 (99–177) minutes, the reconstruction time was 6.5 ± 2.9 (3–14) minutes, the electrophysiologic study time was 10.4 ± 3.4 (6–20) minutes, and the mapping time was 11.7 ± 8.3 (3–36) minutes. No complications occurred. Radiofrequency ablation of arrhythmias without DSA is effective and feasible under the guidance of the Carto 3 mapping system. However, the electrophysiology physician must have sufficient experience, and related emergency measures must be present to ensure safety.

## 1 Introduction

Arrhythmia, including paroxysmal supraventricular tachycardia (PSVT), frequent ventricular premature contractions (VPCs), VT, and atrial fibrillation (AF), is a disease that is frequently evaluated in clinical studies. The main treatment methods for arrhythmia include drugs and/or catheter radiofrequency ablation, and catheter ablation is the main treatment method for arrhythmia in clinical practice. In fact, most cases of arrhythmia in China and abroad are treated in digital subtraction angiography (DSA) operating rooms that provide X-ray guidance for cardiac electrophysiologic examination and radiofrequency ablation treatment.^[1–3]^ X-rays are well known to be associated with radiation risk. Prolonged exposure to X-rays can increase the risk of cancer and other hazards to patients and medical staff.^[1,2]^ Moreover, medical staff members experience increased fatigue and joint load during the operation due to wearing heavy lead clothing for long durations.^[2]^ In addition, X-ray fluoroscopy navigation requires the use of expensive, large DSA machines and specially designed operating rooms, which have become the primary limiting factors of radiofrequency catheter ablation for arrhythmia.

Due to the rapid development of electrophysiologic mapping technology, many desktop computer 3-dimensional (3D) navigation systems that can reconstruct the vascular structure of the heart are currently available; these systems may help avoid X-ray radiation exposure. Non-fluoroscopic 3D mapping systems have been developed to guide mapping and ablation during electrophysiologic procedures, especially for right-sided ablation of supraventricular arrhythmias.^[2,4–7]^ Furthermore, several studies have reported that radiofrequency ablation of arrhythmia can be safely and successfully performed with a zero-fluoroscopy or a minimally fluoroscopic approach (MFA).^[1–4,8,9]^ Even in paroxysmal AF ablation, using only a single-contact force-assisted catheter without X-ray exposure has been found to be both safe and effective.^[10]^

However, no reports on the use of this technique in operating rooms without DSA are available. Our hospital has 4 years of experience in radiofrequency ablation of arrhythmia with the zero-fluoroscopy approach. We have treated more than 400 cases of arrhythmia without serious complications, and related articles have been published.^[3]^ Therefore, this study was designed to investigate the clinical efficacy and safety of catheter ablation for common arrhythmia under the guidance of the Carto 3 mapping system in an operating room without DSA.

## 2 Objectives and methods

### 2.1 Objective of the study

The study was approved by the ethics committee of the Second People's Hospital of Jiedong District and Guangning County People's Hospital in Guangdong Province. Patients undergoing radiofrequency ablation treatment for arrhythmias at from March to October 2017 were recruited. The inclusion criterion was as follows: electrocardiogram (ECG) and/or 24-hour Holter recording showing common arrhythmias, including VPCs, Wolff–Parkinson–White (WPW) syndrome, PSVT, and typical atrial flutter (AFL). All patients exhibited arrhythmias that were resistant to a variety of antiarrhythmic drugs or showed long-term drug intolerance. Additionally, routine inspection by ECG, chest X-ray, and echocardiography had to show that organic heart disease was not present.

### 2.2 Methods

#### 2.2.1 Enrolled patients

**Figure 1:**
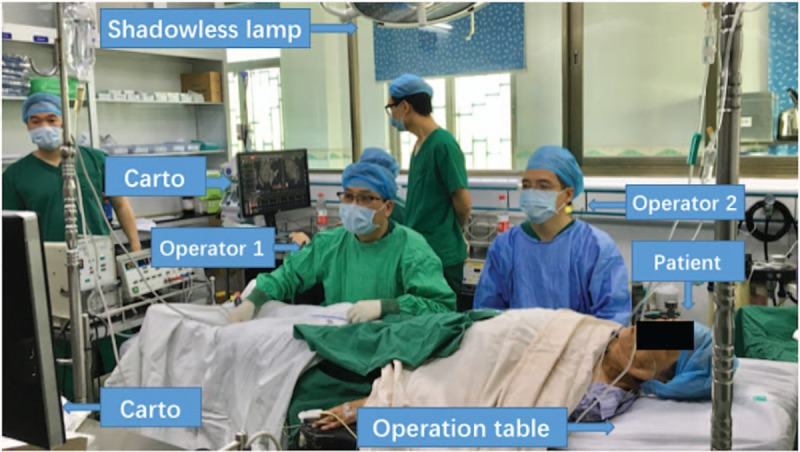
The setup of the operating room.

Antiarrhythmic drugs were discontinued for at least 5 half-lives, and signed informed consent was obtained before the operations. All patients underwent electrophysiologic examination to confirm the type and nature of their arrhythmias. Catheter ablation was performed in the operating room of the Second People's Hospital of Jiedong District and the Guangning County People's Hospital in Guangdong Province. The operating room setup is shown in Figure 1.

#### 2.2.2 Cardiac reconstruction

Cardiac reconstruction is the cornerstone of the entire operation for zero-fluoroscopy ablation. The patients were fasted before the operation. After local anesthesia was applied, the right femoral vein was punctured, and a long guide wire was advanced upward. Then, a long 8.5F preface sheath was inserted. The Carto 3 mapping system (Biosense Webster, Inc, Diamond Bar, CA) was used for cardiac reconstruction. The ablation catheter (SmartTouch, ST; Biosense Webster, Inc) was advanced into the right ventricle through the sheath for anatomical reconstruction. The corresponding anatomical features of the heart, including the superior vena cava, inferior vena cava (IVC), His area, tricuspid valve annulus, right ventricular outflow tract and coronary sinus (CS), were carefully marked. After positioning all diagnostic catheters, a standard electrophysiologic study (EPS) was performed followed by ablation when appropriate. In cases of left chamber arrhythmic substrates, the ablation catheter was inserted through the right femoral artery, and anatomical reconstruction was performed. The ablation catheter was inserted into the artery for retrograde mapping. When the catheter reached the aortic sinus, the root model was carefully constructed, especially the 3 coronary aortic sinuses and the site of the coronary artery.^[3]^ After the aortic sinus model was completed, the head of the catheter was mildly kicked, pushed forward and rotated so that the catheter could cross the valve into the left ventricle. The left side of the His point should be carefully marked to avoid injury. In addition, respiratory gating technology was used to minimize the effect of patient respiration on the location of the catheters in the 3D system. The location of the catheter in the heart cavity was determined by evaluating the catheter head-end potential and the position of the catheter in the 3D system. Intracardiac electrograms were filtered from 30 to 500 Hz and measured at a sweep speed of 100 mm/s.

#### 2.2.3 EPS, mapping, and ablation

The source of right heart arrhythmia, such as atrioventricular nodal re-entrant tachycardia (AVNRT), type B WPW syndrome, typical AFL, and VPCs, originated from the right ventricular outflow tract (RVOT). Therefore, we performed right heart reconstruction according to the above-mentioned method. Then, the quadrupole electrode and the CS electrode were inserted and positioned for electrophysiologic examination. The types of arrhythmias were confirmed by the appropriate procedures, graded stimuli, and drug stimulation. Detail mapping and ablation were performed using the ablation catheter. The procedure time was defined as the time between the patient entering and exiting the operating room.

The mapping methods were as follows. For VPC mapping, activation mapping and pacing mapping were used. Activation mapping is based on the local potential recorded by the ablation catheter and the onset of premature QRS waves, which are measured point by point until the earliest activation point is found. The target is a local potential ahead of the premature QRS wave by at least 20 milliseconds and a unipolar wave that is present as QS wave. Then, pacing mapping was performed at the earliest activation sites. When at least 11 of the 12-lead ECG patterns match the VPCs, the site is referred to as the ablation target. For AVNRT mapping, the His cloud is marked first as the slow path region is below it. The slow pathway improvement method was used to ablate AVNRT. WPW syndrome mapping was defined as sinus rhythm during the earliest ventricular activation preceding the delta wave on the surface by at least 20 milliseconds; otherwise, ventricular pacing at the earliest atrial activation was ablated. If the pre-excitation wave disappeared within 10 seconds, then the corresponding target position was marked in the Carto 3 mapping system, and we continued to consolidate the ablation. Otherwise, detail mapping continued until the correct target was confirmed. The above ablations were performed in temperature control mode with 10 to 20 g of pressure at 30 W and 55°C. Atrial flutter was treated by linear ablation from the tricuspid valve to the IVC. The ablation was performed in cold saline mode with 10 to 20 g of pressure at 30 to 35 W and 43°C; the saline flow rate was 17 mL/min. The criteria of success were as follows. Disappearance of VPCs 30  minutes after intravenous isoproterenol administration indicated successful ablation. Ablation of AVNRT was considered successful if adequate changes in electrophysiologic parameters were achieved during the procedure and if no supraventricular tachycardia could be induced after the last ablation pulse, either under basal conditions or under isoprenaline intravenous infusion. Successful ablation of WPW syndrome was defined as disappearance of pre-excitation and demonstration of blockade after intravenous adenosine treatment. AFL ablation was considered effective when bidirectional isthmus blockade was achieved.

#### 2.2.4 Follow-up

After the ablation procedure, the patients were hospitalized overnight with telemetry monitoring. Data on symptom recurrence, ECGs and 24-hour Holter monitoring were used to assess the success rate of ablation during the follow-up.

#### 2.2.5 Statistical analysis

Continuous data are described as the mean ± standard deviation. Data were analyzed using the SPSS statistical package for Windows version 20.0 (SPSS Inc, Chicago, IL).

## 3 Results

### 3.1 Baseline characteristics

A total of 20 patients were included in this study, including 12 males. The average age was 51.3 ± 17.2 (19–76) years. Nine cases of AVNRT, 7 cases of frequent VPCs, 3 cases of WPW syndrome, and 1 case of typical AFL were included. All arrhythmias were successfully ablated. The procedure time was 127.0 ± 21.0 (99–177) minutes, the reconstruction time was 6.5 ± 2.9 (3–14) minutes, the EPS time was 10.4 ± 3.4 (6–20) minutes, and the mapping time was 11.7 ± 8.3 (3–36) minutes (Table 1). Additionally, no complications occurred. The follow-up time was 4.2 ± 2.3 (1–8) months, and no recurrence of operation-related arrhythmia was observed.

**Table 1:**
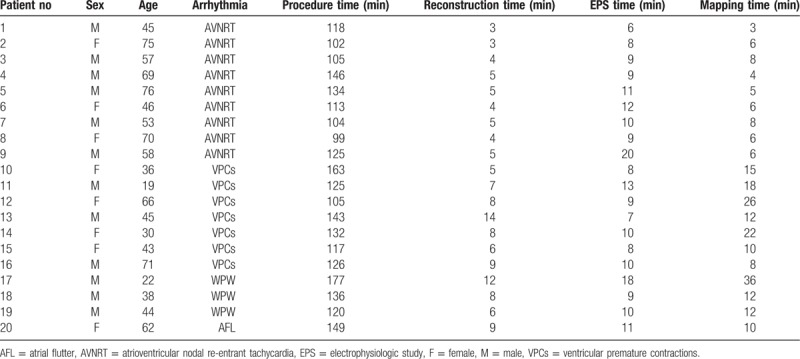
Main parameter of the study population.

The following are representative cases. Case 1: a 22-year-old male experienced recurrent cardiac palpitations for more than 2 years without syncope, and each episode required in-hospital medication administration. This time, catheter ablation was required. The 12-lead ECG indicated type B WPW syndrome (Fig. 2  A). Preoperative echocardiography indicated normal cardiac structure and function, and the left ventricular ejection fraction (LVEF) was 58%. After regular reconstruction and electrophysiologic examination, the arrhythmia type was determined. In addition, the ablation catheter was measured at 12 o’clock relative to the tricuspid valve, and the potential was 35 milliseconds earlier than the delta wave (Fig. 2  B). A 5-second ablation was performed at that point, the potential atrioventricular wave was separated, and the ablation continued to be consolidated. Subsequently, a change was observed in the ECG, which suggested the presence of another left accessory pathway. The model was then constructed by delivering the ablation catheter to the aortic root. The catheter was advanced to the left ventricular side of the mitral valve. The coronary artery electrode was used as the reference to identify the earliest ventricular excitation point during pre-excitation. The catheter potential was 21 milliseconds ahead of the surface delta wave at the white spot (Fig. 2  C). After ablation, the delta wave disappeared within 3 seconds. The ablation was consolidated at the site 4 times. After half an hour, electrophysiologic examination showed that the accessory pathways were blocked. Figure 2  D shows the fusion diagram of the Carto 3 mapping. The overall procedure time was 177  minutes. The postablation ECG was shown in Figure 2  E. At the 5-month follow-up, the patient did not exhibit symptoms, and the ECG was normal.

**Figure 2:**
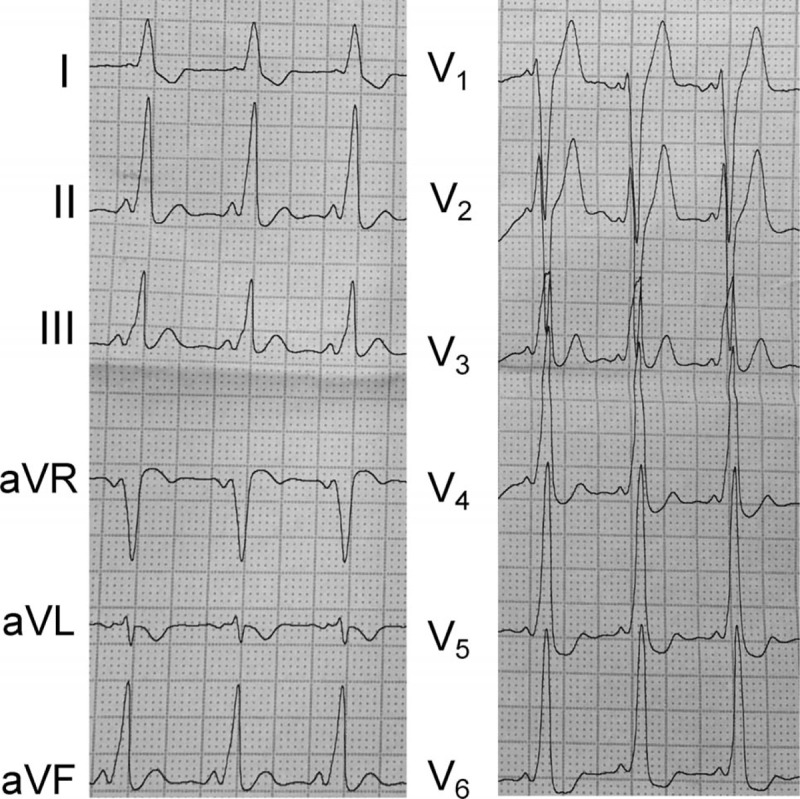
(A) Electrocardiogram (ECG) indicating type B pre-excitation syndrome in case 1. (B) The ablation catheter mapped the potential preceding the delta wave by 35 milliseconds. The yellow spot denotes the location of His, the blue dots denote the earliest area, and the red spots denote the ablation points. The pink points indicate the coronary sinus, and the green line indicates the tricuspid valve annulus. (C) The ablation catheter mapped the potential preceding the delta wave by 21 milliseconds. The yellow point indicates the left His location, the white spots indicate the earliest areas, and the red spots indicate the ablation points. (D) 3D image after merging. (E) The postablation ECG of case 1. LM, left main; LCC, left coronary cusp; RCC, right coronary cusp; NCC, noncoronary cusp.

**Figure 2 (Continued):**
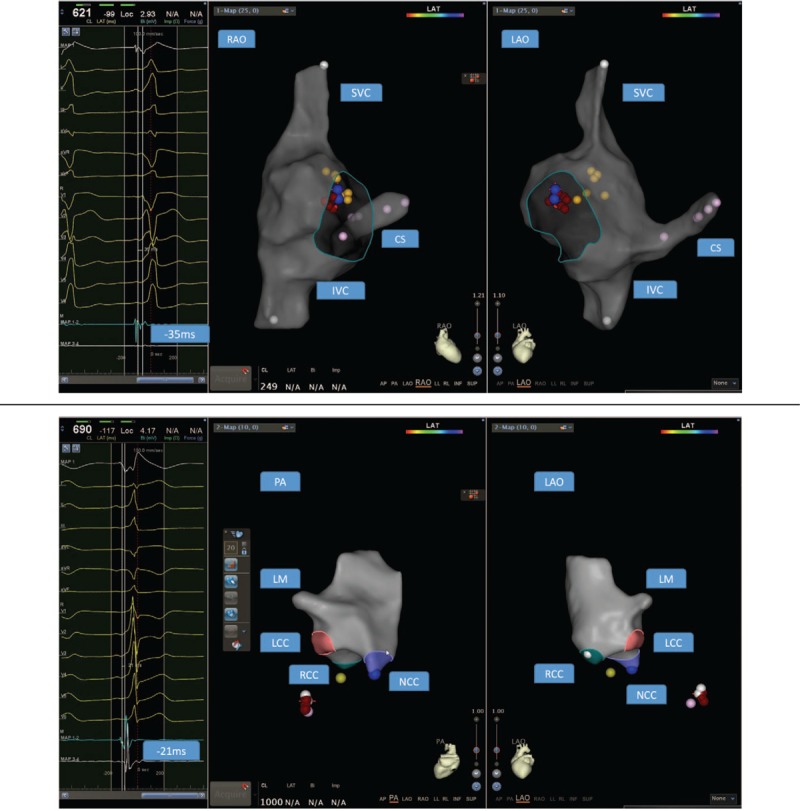
(A) Electrocardiogram (ECG) indicating type B pre-excitation syndrome in case 1. (B) The ablation catheter mapped the potential preceding the delta wave by 35 milliseconds. The yellow spot denotes the location of His, the blue dots denote the earliest area, and the red spots denote the ablation points. The pink points indicate the coronary sinus, and the green line indicates the tricuspid valve annulus. (C) The ablation catheter mapped the potential preceding the delta wave by 21 milliseconds. The yellow point indicates the left His location, the white spots indicate the earliest areas, and the red spots indicate the ablation points. (D) 3D image after merging. (E) The postablation ECG of case 1. LM, left main; LCC, left coronary cusp; RCC, right coronary cusp; NCC, noncoronary cusp.

**Figure 2 (Continued):**
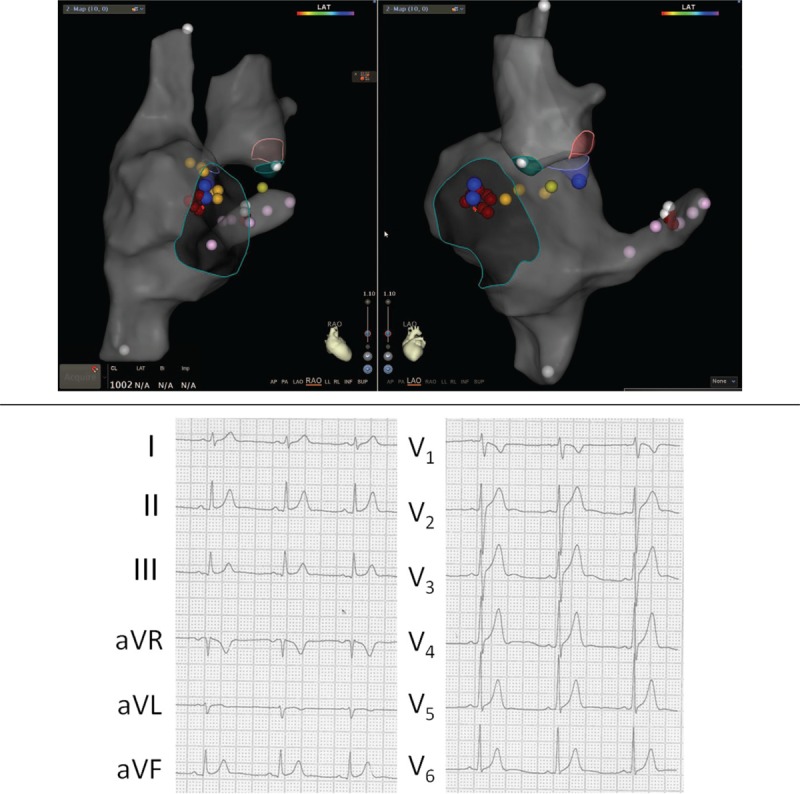
(A) Electrocardiogram (ECG) indicating type B pre-excitation syndrome in case 1. (B) The ablation catheter mapped the potential preceding the delta wave by 35 milliseconds. The yellow spot denotes the location of His, the blue dots denote the earliest area, and the red spots denote the ablation points. The pink points indicate the coronary sinus, and the green line indicates the tricuspid valve annulus. (C) The ablation catheter mapped the potential preceding the delta wave by 21 milliseconds. The yellow point indicates the left His location, the white spots indicate the earliest areas, and the red spots indicate the ablation points. (D) 3D image after merging. (E) The postablation ECG of case 1. LM, left main; LCC, left coronary cusp; RCC, right coronary cusp; NCC, noncoronary cusp.

Case 2: A 71-year-old male required radiofrequency ablation treatment due to recurrent palpitations for more than 1 year which were poorly controlled by medication. The ECG indicated frequent VPCs (Fig. 3 A), and the VPC burden was 35% as determined by the Holter monitor. Preoperative echocardiography indicated normal cardiac structure and function, with an LVEF of 52%. According to the ECG, the VPCs may have originated from the aortic sinus. The right femoral artery was then punctured, and reconstruction was performed as described above. The catheter potential preceded the ECG at the left coronary cusp (LCC) by 55 milliseconds, and the pacing ECG was almost the same as the spontaneous VPCs (Fig. 3 B and C). Ablation was performed at this point and was successful. After intravenous infusion of isoproterenol, VPCs were no longer observed. Figure 3 D shows the Carto image. The total procedure time was 126  minutes. The postablation ECG was shown in Figure 3 E. The patient did not exhibit symptoms and the ECG was normal at the 4-month follow-up.

**Figure 3:**
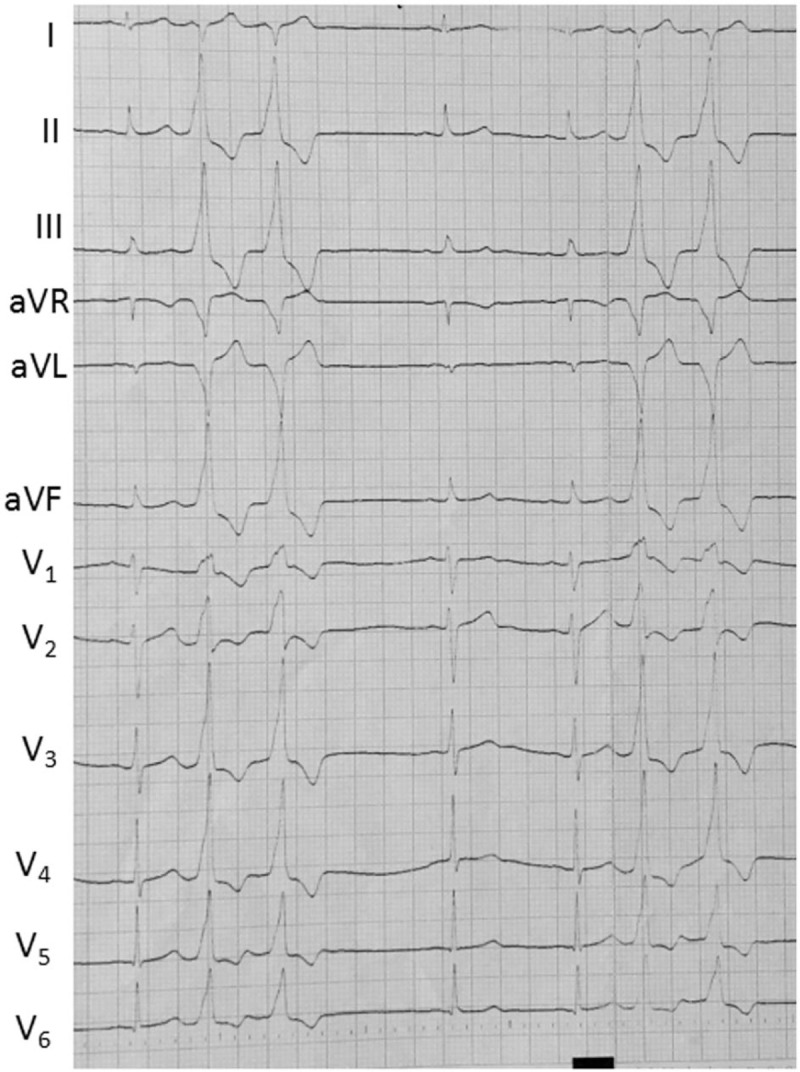
(A) Electrocardiogram (ECG) suggesting frequent ventricular premature contractions (VPCs). (B) The ablation catheter mapped the potential preceding the delta wave by 55 milliseconds. (C) The pacing ECG was consistent with spontaneous VPCs. (D) 3D mapping shows the LL and LAO 45° positions: the red spot indicates the earliest excitation spot, and the white spot indicates the LCC position. (E) The postablation ECG of case 2.

**Figure 3 (Continued):**
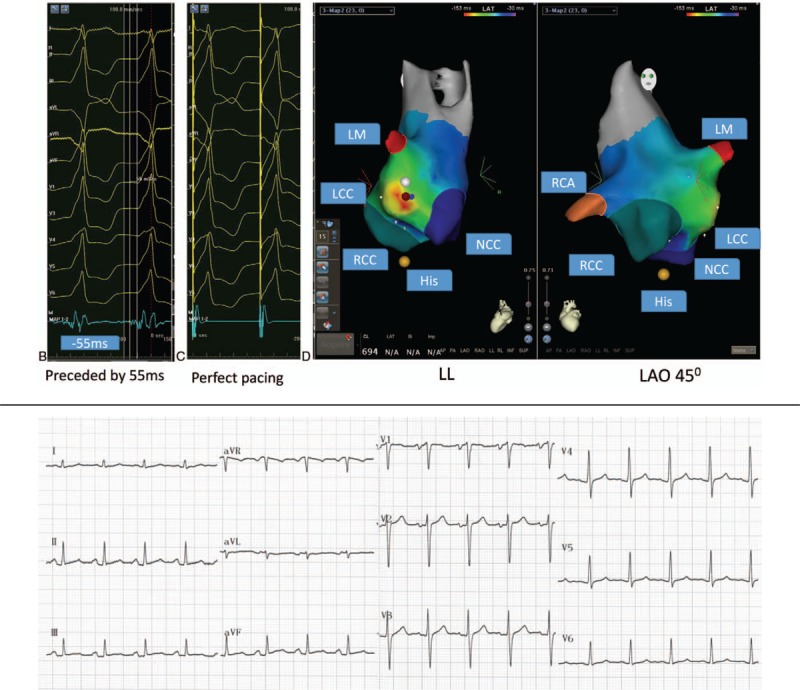
(A) Electrocardiogram (ECG) suggesting frequent ventricular premature contractions (VPCs). (B) The ablation catheter mapped the potential preceding the delta wave by 55 milliseconds. (C) The pacing ECG was consistent with spontaneous VPCs. (D) 3D mapping shows the LL and LAO 45° positions: the red spot indicates the earliest excitation spot, and the white spot indicates the LCC position. (E) The postablation ECG of case 2.

## 4 Discussion

### 4.1 Main finding

To our knowledge, this is the first case series of tachycardia ablation procedures performed without DSA backup. Our study indicates that radiofrequency ablation can be performed in an operating room without DSA or multiple physiologic instruments under the guidance of the Carto 3 mapping system. The arrhythmias ablated in the present study included ANVRT, WPW syndrome, typical AFL, and VPCs originating from the RVOT and aortic sinus, all of which were successfully ablated without complications. Additionally, selecting an approach without DSA also affects the ionizing radiation exposure of the medical staff. Some studies have also reported on catheter ablation of AF without fluoroscopy and demonstrated the safety, feasibility, and effectiveness of minimal fluoroscopic ablation in this patient population^[10–13]^; however, we did not consider patients with AF in this population.

### 4.2 Rationale of cardiac radiofrequency ablation in a non-DSA room

Radiofrequency ablation is one of the most effective treatments for arrhythmia. Traditional radiofrequency ablation is performed under X-ray guidance in an operating room equipped with DSA. However, X-rays are potentially harmful to patients and doctors. Over the past decade, various measures and technologies, such as the contact force-sensing catheter,^[14]^ Carto Univu mapping systems,^[15]^ and the real-time remote magnetic catheter navigation system,^[16]^ have been used to improve the procedural outcomes of fluoroscopy.^[8]^ With these improvements, procedural and fluoroscopic times have progressively decreased.^[17]^ Zero or near-zero fluoroscopic ablation of arrhythmias is gradually being introduced in clinical practice.^[8]^ Recently, many studies have reported that radiofrequency ablation under zero-fluoroscopy guidance using a 3D mapping system is safe and effective.^[1,2,4–10,15,18]^ Our center has also contributed related reports to the literature.^[3]^ Most centers require intracardiac echocardiography (ICE) guidance,^[11,15]^ Carto Univu systems^[1,15]^ or pressure catheters^[3,5,6,8,10]^ for zero-fluoroscopy radiofrequency ablation. In addition, DSA operating rooms require multiple conductive physiologic systems for cardiac electrophysiology operations. However, many primary hospitals cannot achieve these conditions, thus restricting the use of cardiac electrophysiologic intervention.

The Carto 3 mapping system can perform heart geometric reconstruction in detail via fast automatic reconstruction. At the same time, the system can display the real-time position and course of the ablation catheter in the heart cavity. Thus, scientific and technologic improvements have enabled radiofrequency ablation without fluoroscopy, which has been demonstrated in the clinical setting. In addition, the ablation model uses a safer temperature control mode, which reduces the risk of complications associated with the ablation process. Therefore, intracardiac electrophysiologic examination and cardiac radiofrequency ablation have become a reality in operating rooms without DSA.

### 4.3 Economic considerations

The economic considerations for the health security system are becoming increasingly important. In terms of cost-effectiveness, in general, the Carto system was much more expensive than conventional ablation. However, China has unique health system protocols. First, medical security is divided into different grades, and patients can receive greater reimbursement when they are treated in primary hospitals. Second, ICE was not used in our study, and only an ST catheter was used, which can reduce costs substantially. Lastly, Casella et al proved that the MFA clearly has clinical benefits for both patients and medical staff as it decreases the risk of cancer due to radiation exposure.^[1]^ No fluoroscopy was used in this study, which may be more important in terms of the health of patients and medical staff rather than money. Regarding success rates and complications rates, many studies have reported that radiofrequency ablation with the zero-fluoroscopy approach or the MFA was not significantly different compared with conventional ablation.^[1–4,8,9]^ In our study, the success rate was 100% and no major complications occurred.

### 4.4 Clinical implications

The findings of this study may have the following clinical implications: First, with additional improvements in 3D mapping systems and electrophysiologic examination methods, experienced doctors may be able to perform electrophysiologic procedures at the bedside or in intensive care units, thus providing a new vision for radiofrequency ablation. More importantly, cardiac radiofrequency ablation can be performed in primary hospitals without expensive, large X-ray machines or multiple conductive physiologic instruments, thus reducing the need for multiple family members to accompany patients to a large hospital, which can also reduce medical and health care expenses. Therefore, successful development of zero-fluoroscopy ablation methods will allow broad application prospects in the future. Second, similar to previous zero-fluoroscopy studies,^[3,5,6,8,10]^ ST catheter application was essential for performing mapping and ablation in this study. Soft manipulation and accurate reconstruction and mapping enable successful completion of the operation. Third, the medical team does not need to wear heavy lead clothes when X-ray radiation is not used, which can significantly reduce the load- and temperature-related discomfort experienced by surgical teams in China and abroad.^[2]^ The zero-fluoroscopy method is more beneficial for patients, especially pregnant women and old and weak patients.^[1,4–9]^

In summary, cardiac EPS and ablation can be performed by experienced electrophysiology physicians with the guidance of a 3D mapping system. However, since surgery still poses a certain level of risk, a small C arm or cardiac ultrasound should be present in the operating room for evaluation. Therefore, if conditions change, we can quickly evaluate the changes and formulate the appropriate response.

### 4.5 Study limitations

First, this was a nonrandomized study. The selection of arrhythmias for ablation was determined by the operator; therefore, a bias is possible depending on the preference and experience of the operator. Second, few patients were enrolled in this study; thus, this study was not large enough to establish a firm conclusion. Therefore, additional well-controlled randomized trials with larger patient cohorts that further assess ablation of arrhythmias without DSA are required to validate our findings.

## 5 Conclusion

Radiofrequency ablation of arrhythmias without DSA is effective and feasible under the guidance of the Carto 3 mapping system. However, electrophysiology physicians must have sufficient experience, and related emergency measures must be present to ensure safety.

## Author contributions

**Data curation:** Xingfu Huang, Yanjia Chen, Liwei He, Xiaojiang Deng, Yongsheng Wang, Rucheng Li.

**Formal analysis:** Dingli Xu, Jian Peng.

**Investigation:** Xingfu Huang, Zheng Huang, Liwei He, Shenrong Liu.

**Writing – original draft:** Xingfu Huang.

**Writing – review & editing:** Xingfu Huang, Dingli Xu, Jian Peng.
